# Environmental aridity driving latitudinal pattern of biomass allocation fractions in root systems of 63 shrub species in dry valleys

**DOI:** 10.1002/ece3.70091

**Published:** 2024-08-07

**Authors:** Yu Yang, Zilong Wang, Weikai Bao, Ning Wu, Hui Hu, Tinghui Yang, Xiaojuan Li, Deborah Traselin Nkrumah, Fanglan Li

**Affiliations:** ^1^ Chengdu Institute of Biology Chinese Academy of Sciences Chengdu China; ^2^ University of Chinese Academy of Sciences Beijing China; ^3^ Present address: Chengdu Institute of Biology, Chinese Academy of Sciences No. 23, Qunxian Nan Road Chengdu 610213 Sichuan China

**Keywords:** absorptive root, biomass allocation, dry valley, environmental factor, latitudinal pattern

## Abstract

Fine roots and absorptive roots play key roles in acquiring resources throughout soil profiles and determining plant functions along environmental gradients. Yet, the geographical pattern of carbon allocation in fine roots, particularly in absorptive roots, and their relations with plant sizes and evironment are less understood. We sampled 243 xerophytic shrubs from 63 species distributed along the latitudinal gradient (23°N to 32°N) in dry valleys of southwest China and synthetically measured biomass fractions of plant organs, especially fine roots and absorptive roots (1st to 3rd root order). We identified latitudinal patterns of biomass allocation fractions of organs and their relationships with plant sizes and environmental factors. The latitudinal patterns of both absorptive root and fine‐root fractions followed weak unimodal distributions; stem biomass fraction increased with the latitude, while the leaf biomass fraction decreased. The fraction of fine‐root biomass had negative relationships with plant height and root depth. The fractions of root, fine root, and absorptive root biomass were largely explained by soil moisture. Furthermore, fraction of fine‐root biomass increased in a relatively humid environment. Overall, soil moisture was the most important factor in driving latitudinal patterns of biomass fraction. Our study highlighted that functional redistribution of root system biomass was the critical adaptive strategy along a latitudinal gradient.

## Graphical abstract

The latitudinal patterns of both absorptive root fraction and fine‐root fraction followed unimodal distributions. Soil moisture was the most important factor in driving latitudinal patterns of biomass fraction.

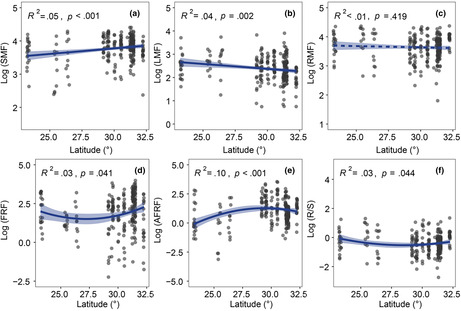

## 1 INTRODUCTION

Biomass allocation to different organs or components of plant individuals varies with environmental change (Litton et al., 2007; McConnaughay & Coleman, 1999; Poorter et al., 2012). The biomass fraction of different plant components reflects functional strategy in resource acquisition and, therefore, determines terrestrial carbon storage (Freschet et al., 2018; Umana et al., 2021). The optimal allocation theory predicts that plants would allocate a greater proportion of biomass to the organs, which could capture more limiting resources in a resource‐poor environment (Bloom et al., 1985; Poorter et al., 2015; Qi et al., 2019). For example, plants prefer to allocate more biomass to their roots to acquire more water and nutrients under cold and dry conditions (Jevon & Lang, 2022; Puglielli et al., 2021). Fine roots are key in absorbing soil resources, which are thus sensitive to environmental changes (Pregitzer et al., 2002). In particular, absorptive roots showed a stronger response to environmental conditions than other functional components (Da et al., 2023). Pierick et al. (2022) and Zadworny et al. (2021) thought that the importance of absorptive roots should not be ignored when studying the function of roots as a whole. However, previous studies on biomass allocation did not pay enough attention to the critical functional parts of root system, such as fine roots and absorptive roots (Ma et al., 2021; Poorter et al., 2015; Reich et al., 2014).

At geographical scale, biomass allocation fractions have diverse patterns in different climatic regions, especially in belowground parts. De Frenne et al. (2013) found that root biomass fraction increased along the increasing latitude, and Finér found no obvious change of fine root biomass at the latitude from 60° N to 69° N. Plants would have more biomass in absorptive roots at higher elevation (Pierick et al., 2022). Usually, plants would increase their biomass fractions of roots and/or leaves to facilitate the acquisition of water and nutrients under resource‐limiting conditions (Ma et al., 2021; Markesteijn & Poorter, 2009). Living in a cold climate, plants also tend to allocate more biomass to absorptive roots to strengthen the resource acquisition from soils (Reich et al., 2014; Zadworny et al., 2021). However, biomass allocation is determined by not only climate but also soil properties at local scale. Yan et al. (2016) also found that climate exerted an influence on the biomass allocation of stems but was not as important as soil nutrients. Pierick et al. (2022) found that plants allocated more biomass to absorptive roots to compensate for the resource shortage while soil conditions were unfavorable. Plants increase the biomass of their roots or leaves to compensate for nutrient absorption loss in a challenging environment (Rehling et al., 2021). And some forests allocated less biomass to roots but more to the leaves while they grew in low nitrogen soil (Umana et al., 2021). Hu et al. (2021) reported that biomass allocations of *Artemisia vestita* and *Bauhinia brachycarpa* were influenced by multiple factors, including soil physical properties which might affect soil moisture and then influence the pattern of biomass allocation in plants. However, so far, there still has not been enough evidence to reveal the comprehensive mechanism of major environmental drivers such as climatic and edaphic factors in determining the rooting strategy of plants in responding to environmental stress in arid or semi‐arid ecosystems.

In a community, shorter species typically invest relatively more biomass in their leaves and roots than in stems (Dolezal et al., 2020; Poorter et al., 2012, 2015). The vertical and horizontal extensions of plants partly define their capacity and preference in competition for resources. For example, more biomass allocated to stems was usually favored for capturing more light resources (Poorter et al., 2015), but deep rooting strategy or expanding lateral roots was adopted by the species living in low water availability (Schenk & Jackson, 2002; Zhou et al., 2020). Positive co‐variation between root biomass fraction and rooting depth could be beneficial to balance the investments of plants in root architecture and then effectively exploit soil water or nutrients (Freschet et al., 2018). With the change in environmental conditions, such as water availability belowground being no longer limited, plants could readjust their adaptive strategy accordingly, increasing above‐ground biomass while reducing the investment in root systems (Tumber‐Dávila et al., 2022). Therefore, the trade‐off relationship between expanding range of individuals and their capacity for resource acquisition is likely to play an important role in optimizing the strategy of plants acquiring soil resources.

Latitudinal gradient indicates a varying sequence of environmental factors, such as water and heat, which could result in the adaptive change of plant biomass allocation at a spatial scale (Frenne et al., 2013; Ostonen et al., 2017). In order to study the latitudinal pattern of biomass allocation and its drivers in dry valleys in mountains of China Southwest, we selected 63 native shrub species along a 1000 km latitudinal gradient. We measured parameters including plant size, biomass fractions of stems, leaves, roots, fine roots, and absorptive roots, as well as soil properties. This study mainly addressed three questions: (1) How does the biomass allocation of different plant components change along the latitudinal gradient of dry valleys? (2) How do the fractions of biomass allocation vary with different plant sizes? (3) How do environmental factors, mainly including climatic and edaphic factors, drive the formation of latitudinal patterns of biomass fractions?

## 2 MATERIALS AND METHODS

### 2.1 Study area

The study areas are the dry valleys located in Sichuan and Yunnan provinces (23°13′53.731″ ~ 32°15′38″ N, 101°9′51.451″ ~ 103°31′20″ E), and belonging to the China Southwest Mountains, one of 36 global biodiversity hotspots defined by IUCN. The dry valleys cover an area of approximately 2.6 × 10^4^ km^2^ along a series of north–south orientated rivers, including Bailong, Minjiang, Dadu, Yalong, Jinsha (upper Yangtze), Lancang (upper Mekong) and their tributaries (Fan et al., 2020). The climate at the bottom of these valleys is generally dry and hot with the mean annual precipitation ranging from 662 to 1350 mm, the mean annual evapotranspiration from 1329 to 3214 mm, and the mean annual temperature from 6.42°C to 24.1°C (Fan et al., 2020). Sparse vegetation and infertile soils covering mountain slopes vary spatially along the latitudinal gradient of these valleys (Li et al., 2023; Wei, 2021; Yang et al., 2023). Due to the seasonal aridity, over 80% rainfall occurring from June to August, xerophytic shrubs are dominant species in vegetation communities, such as *Vitex negundo*, *Dodonaea viscosa*, *Desmodium elegans*, *Bauhinia brachycarpa*, *Cymbopogon distan*, *Euphorbia heterophylla*, *Selaginella mairei*, and *Boea hygrometrica*, presenting a savannah‐like landscape (Wei, 2021).

**FIGURE 1:**
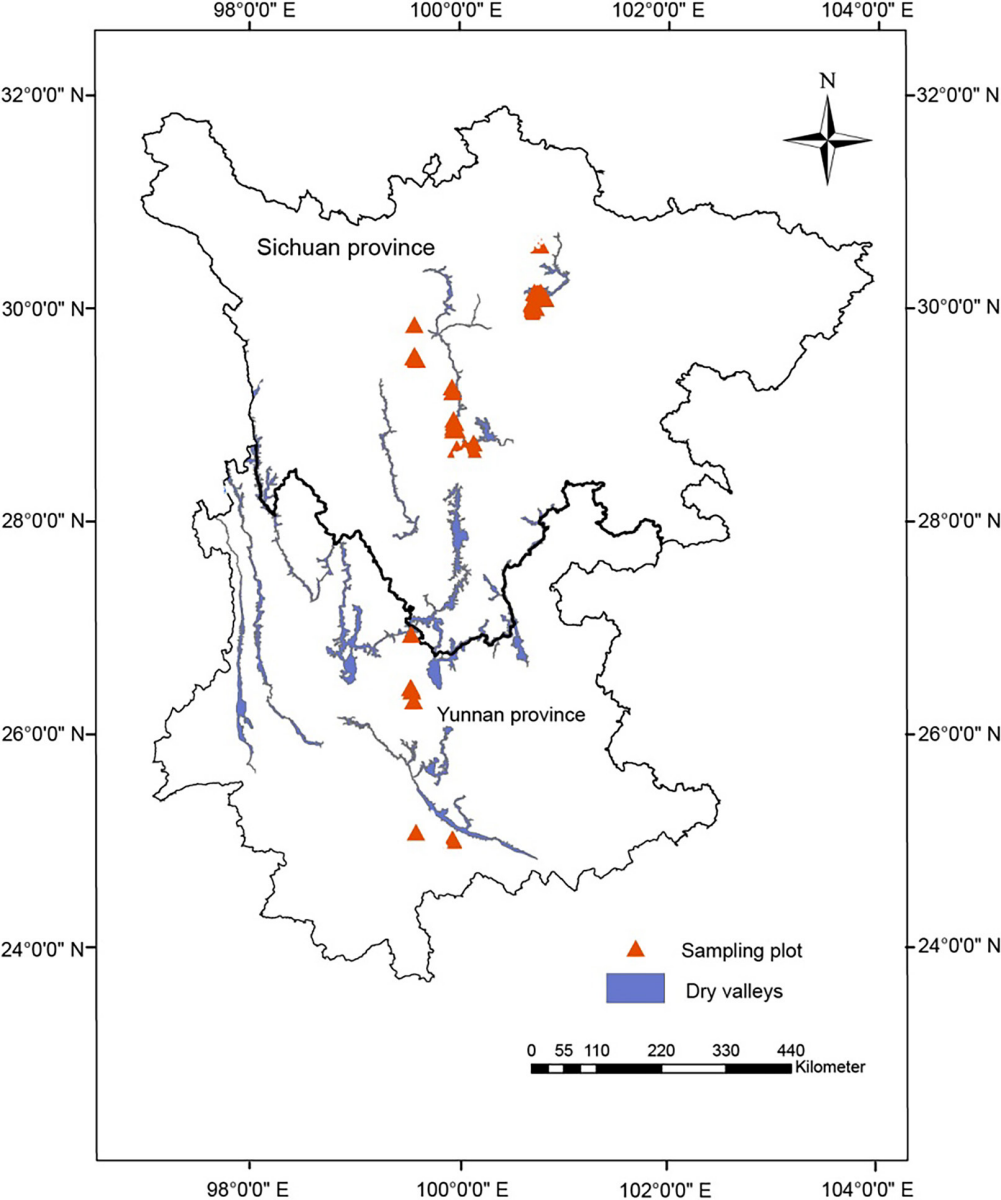
The 92 sampling plot across latitudinal gradient in the dry valleys of Sichuan and Yunnan provinces in southwest China (the sampling range across latitude at 23° N to 32° N).

In July and August of 2019–2020, we selected 92 plots (4 × 6 m) without obvious human disturbance along the dry valleys, distributed at 10 latitudinal sites from 23° N to 32° N (Figure 1; Table S1). Before assessing the biomass of communities, quadrants were used to identify the morphological characteristics of vegetation. Three shrub quadrants (2 × 2 m) were established on the diagonal of each plot, in which all species were identified. Selected randomly one of three quadrats in each plot, all shrub individuals were collected to measure plant size and biomass fraction. In general, 63 species, comprising 243 individuals (Table S2), were measured.

### 2.2 Measurements of plant size and biomass

Plant height, the distance from ground level to the upper limit of individual, was measured with a tape measure (unit: mm, Tumber‐Dávila et al., 2022). Ten representative stems per individual were randomly selected to measure base diameter with a caliper (unit: mm, MarCal 16 U, Germany). A spud was used to explore the maximum extension of root system. The spreading length of lateral roots (LL; unit: cm) was calculated based on the distance of longest lateral root extending horizontally from plant base stem (Schenk & Jackson, 2002). The maximum depth of rooting was defined as the deepest soil layer reached by 95% of fine roots (unit: cm, Schenk & Jackson, 2002).

After measuring plant size, the above‐ground parts of individuals were cut off from the stem base, and then leaves and stems (or branches) were separated manually. The root system was excavated with a shovel and all (or a half for big individuals) of leaves, stems and roots were labeled before being brought back to the laboratory. To minimize the removal damage of samples, we filled back soil in original location. This procedure could ensure the restoration of the original vegetation by using their native soil seed bank and proliferation of nearby shrubs species (Lortie et al., 2018; Shang et al., 2009). Root samples were carefully submerged in water to remove adhering soils in the laboratory. Then root samples were sorted out for biomass measurement, including total roots, fine roots (< 2 mm in diameter), and absorptive roots (1st to 3rd root orders) (McCormack et al., 2015; Pregitzer et al., 2002). These subsamples of leaves, stems (or branches) and roots were dried at 75°C for 48 h in a drying closet (DHG‐9030, Yiheng, China) to determine their dry weights. The biomass allocations to leaf (LMF), stem (SMF), and root (RMF) were calculated based on the dry weight of each organ divided by the total dry weight of each individual. In this study the root‐to‐shoot ratio (R/S) means the ratio of below‐ground to above‐ground biomass; the fine root mass fraction (FRF) is the proportion of fine roots to the total mass of roots; and the absorptive root mass fraction (AFRF) is the proportion of absorbing roots to fine roots.

### 2.3 Soil physicochemical analysis

Eighteen soil cores per plot were collected from topsoil (0–10 cm) and subsoil (10–20 cm) using a stainless‐steel cylinder (5 cm diameter) and pooled to form a composite sample. Visible roots, residues, and stones in soils were removed before homogenizing each sample. All fresh soil samples were transported with cool boxes, sieved through a 2‐mm mesh, and divided into two subsamples. A subsample was stored at −20°C for phospholipid fatty acid (PLFA) extraction, while another was stored at 4°C for the determination of soil physicochemical properties.

Based on previous studies (Wang et al., 2022), some key soil indicators were measured to explore the relationships between biomass allocation and soil properties. The bulk density of soil (BD) was measured by cutting rings (100 cm^3^). In each sampling plot, nine undisturbed soil samples were collected and oven‐dried at 105°C for calculating the bulk density of soil (BD). Soil pH was measured in a suspension sample (1:2.5 w/v soil to water) with a digital pH meter. Inorganic nitrogen (TIN) was calculated as the sum of ammonium and nitrate. The dissolved organic carbon (DOC) was extracted with deionized water (1: 5 w/v of soil to water) and determined using a TOC/TN analyzer (Vario TOC, DKSH, China). The soil cation exchange capacity (CEC) was treated with sodium acetate to saturate with Na^+^. The excess sodium acetate was washed with 95% ethanol or 99% isopropyl alcohol. The exchangeable Na^+^ was exchanged with NH4^+^, and the Na^+^ in the solution was determined by flame spectrophotometry to calculate the exchange capacity of ions (Lu, 1999). Soil particle size distribution (PSD) based on soil texture was measured with a Mastersizer 2000 (Malvern Instruments, Malvern, England).

### 2.4 Climate data

Mean annual temperature (MAT) and mean annual precipitation (MAP) with a spatial resolution of 30 arc‐seconds from 1970 to 2000 were collected from the WorldClim website (https://www.worldclim.org/), and the data were extracted using ArcGIS (ArcGis10.2.2, Esri, USA). Estimates for mean annual potential evapotranspiration (MAE) were obtained from the Global Aridity Index and the Potential Evapotranspiration Climate Database v.2. The aridity index (AI) was calculated by MAP/MAE. Soil moisture data was obtained from the Soil Moisture of China website (Shangguan et al., 2022).

### 2.5 Statistical analysis

**FIGURE 2:**
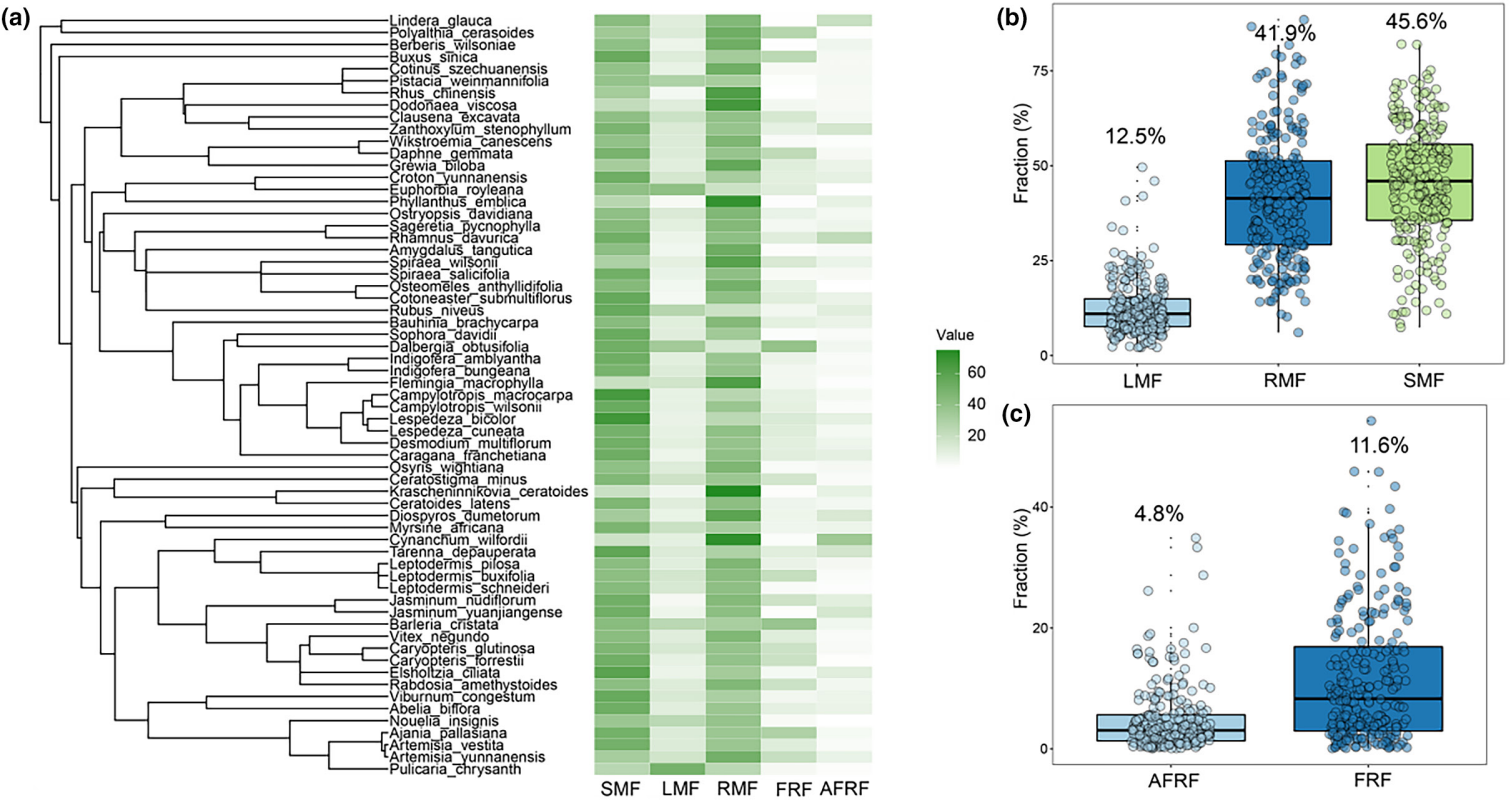
Phylogenetic tree and distributions of biomass allocation fractions of 63 species in dry valleys of southwest China. AFRF, Absorptive root mass fraction in fine‐root system; FRF, Fine‐root mass fraction in root system; LMF, Leaf mass fraction; RMF, Root mass fraction; SMF, Stem mass fraction.

A phylogenetic tree was constructed using the R package “V.PhyloMaker” (Qian & Jin, 2016). This phylogenetic tree showed the evolutionary distance of each species (Figure 2a). Five mean biomass fractions of each species were used in phylogenetic analyses. Phylogenetic signals (Blomberg's K and Pagel's Lambda) of five biomass fractions were tested with package R “phytools” (O'Brien, 2014; Revell, 2012).

Linear regression was used to assess the relationships between latitude and biomass allocation fractions and confirm the latitudinal patterns of each biomass allocation fraction. Principal component analysis (PCA) was used to assess the relationship between biomass allocation fractions and plant sizes with the R package “FactoMineR” (Le et al., 2008). To explore the variation of environmental influence on biomass allocation fractions at individual level, incorporating species as random effects, linear mixed‐effects models were used by package “lme4” (Bates et al., 2015). The hierarchical partitioning method was used to calculate the percentage of variance explained for every environmental factor by package R “glmm.hp” (Lai et al., 2022). The most important factors of hierarchical partitioning were used in the structural equation model to explore the pathway relationships between climate, soil, and biomass allocation fractions along latitudinal gradients using package R “piecewiseSEM” (Lefcheck, 2016). Non‐normal data were natural log‐transformed before analysis. Statistical analyses were performed in R version 4.1.2 (R Core Team, 2020).

## 3 RESULTS

### 3.1 Biomass allocation fractions

In dry valleys, both root and stem biomass allocation fractions of 63 sampled species were approximately three‐fold of their leaf fractions. LMF ranged from 2.1% up to 49.6% with a mean value of 12.5% (Figure 2b). On average, SMF and RMF were 45.5%, and 41.9%, respectively, with the former ranging from 8.4% to 82.0% and the latter from 6.1% to 81.8%. Within the RMF, FRF with a range of 0.07%–54.3% made up 11.6% of the whole, while AFRF with a range of 0.04%–34.9% only accounted to 4.8% in FRF (Figure 2c). This study did not find a significant phylogenetic signal in biomass fractions (Table S3).

**FIGURE 3:**
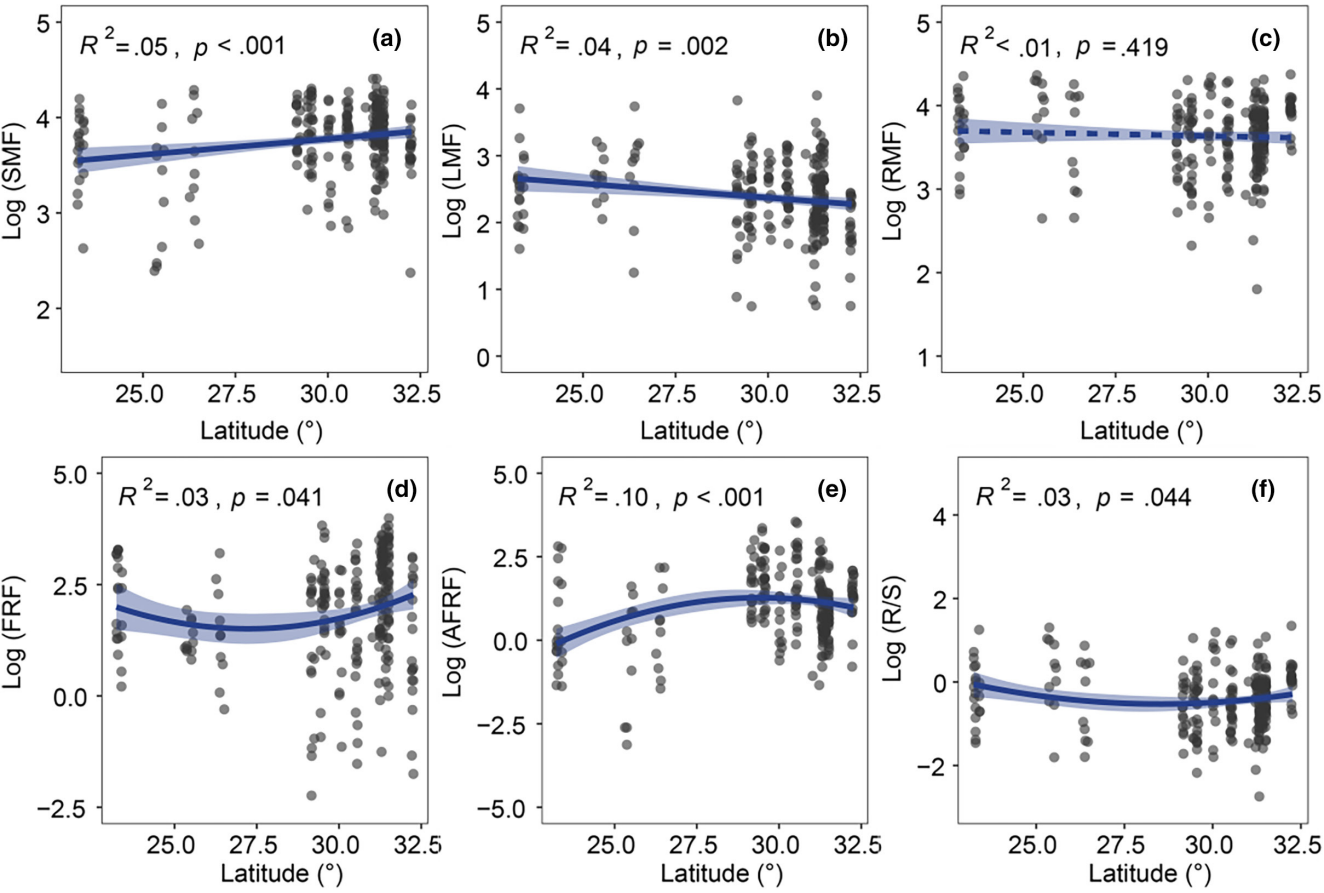
The latitudinal pattern of biomass allocation fractions of different organs in dry valleys of southwest China. AFRF, Absorptive root mass fraction; FRF, Fine‐root mass fraction; LMF, Leaf mass fraction; RMF, Root mass fraction; R/S, Root‐shoot ratio; SMF, Stem mass fraction.

SMF increased with the increasing latitude (Figure 3a), but LMF showed a decreasing trend (Figure 3b). For RMF, there was not a significant change along the latitudinal gradient (Figure 3c). The latitudinal patterns of FRF, AFRF, and R/S were weakly unimodal.

### 3.2 Trade‐off relationships between biomass allocation fractions and plant sizes

**FIGURE 4:**
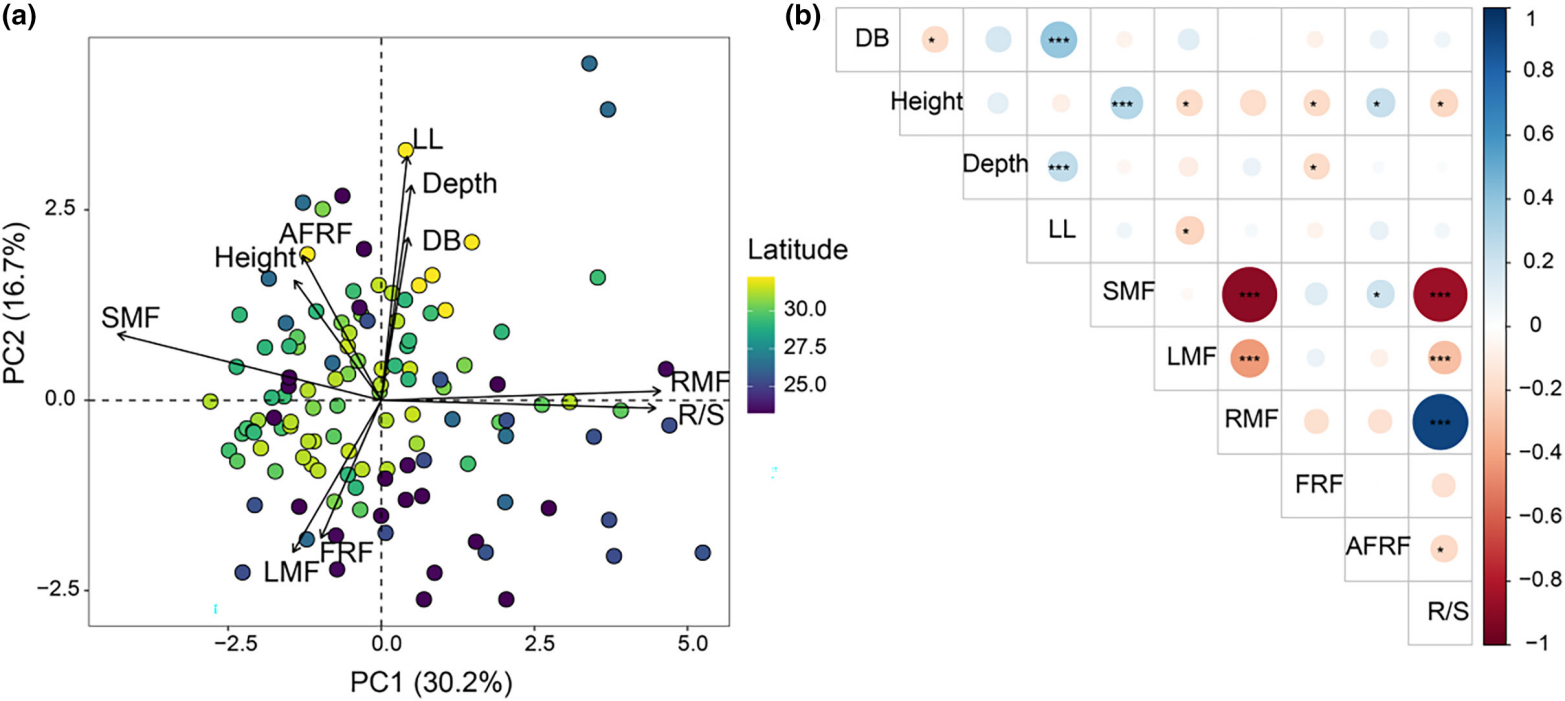
Principal component analysis (PCA) of plant biomass allocation fractions (a) and Pearson's correlations between plant size and mass allocation fractions (b). AFRF, Absorptive root mass fraction; DB, Basal diameter; Depth, Root depth; FRF, Fine‐root mass fraction; Height, Plant height; LL, Lateral root extension length; LMF, Leaf mass fraction; RMF, Root mass fraction; R/S, Root‐shoot ratio; SMF, Stem mass fraction. *p < .05, **p < .01, ***p < .001.

The first principal component axis of PCA represented 30.2% of the total variance, indicating primarily the variations in plant R/S ratio, RMF, and SMF (Figure 4a). The plants with higher SMF tended to have lower RMF and thus a lower R/S ratio. The second principal component axis represented 16.7% of the total variance, indicating rooting depth and the spreading length of lateral roots (Figure 4a). The correlation analysis showed significant positive relationships between stem base diameter, root depth, and lateral root spreading (LL; Figure 4b). However, LMF was closely and negatively associated with LL. FRF had a negative relationship with plant height and root depth. Meanwhile, plant height was negatively related to SMF and AFRF (Figure 4b).

### 3.3 Environmental factors influencing biomass allocation fractions

**FIGURE 5:**
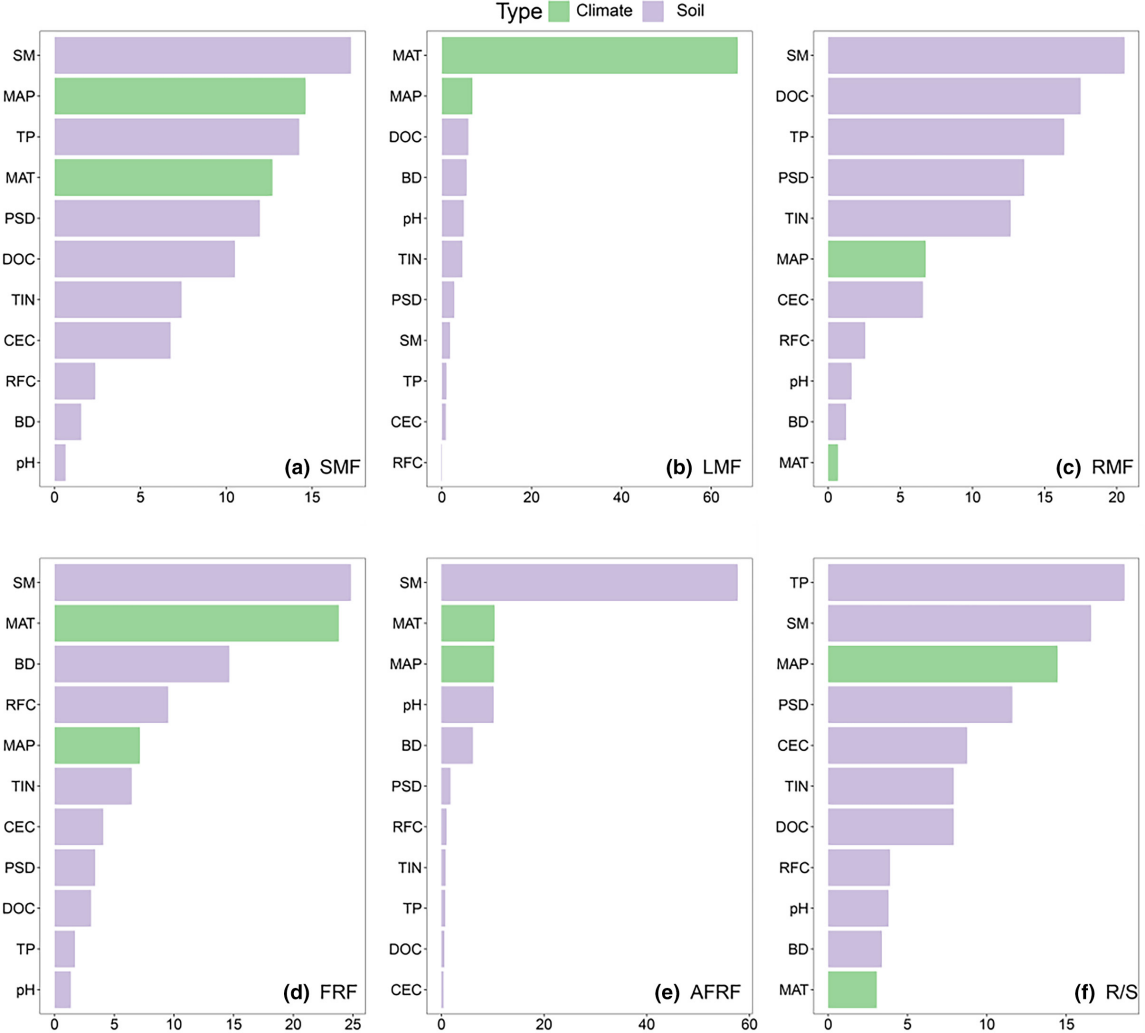
Contribution of climate and soil to the variance of biomass allocations from linear mixed‐effects models based on hierarchical partition. AFRF, Absorptive root mass fraction; BD, Bulk density; DOC, Dissolved organic carbon; FRF, Fine‐root mass fraction; LMF, Leaf mass fraction; MAP, Mean annual precipitation; MAT, Mean annual temperature; pH, Pondus hydrogenii; PSD, Particle size distribution; RFC, Rock fragment content; RMF, Root mass fraction; R/S, Root‐shoot ratio; SM, Soil moisture; SMF, Stem mass fraction; TIN, Total inorganic nitrogen.

The results of hierarchical partition showed that soil moisture was the most important factor impacting the variation of plant biomass fractions except for LMF and R/S (Figure 5). Moreover, the total soil phosphorus content was the most important factor for R/S ratio, while MAT was the dominant factor for explaining the variation of LMF across species along the latitudinal gradient (Figure 5b,f).

**FIGURE 6:**
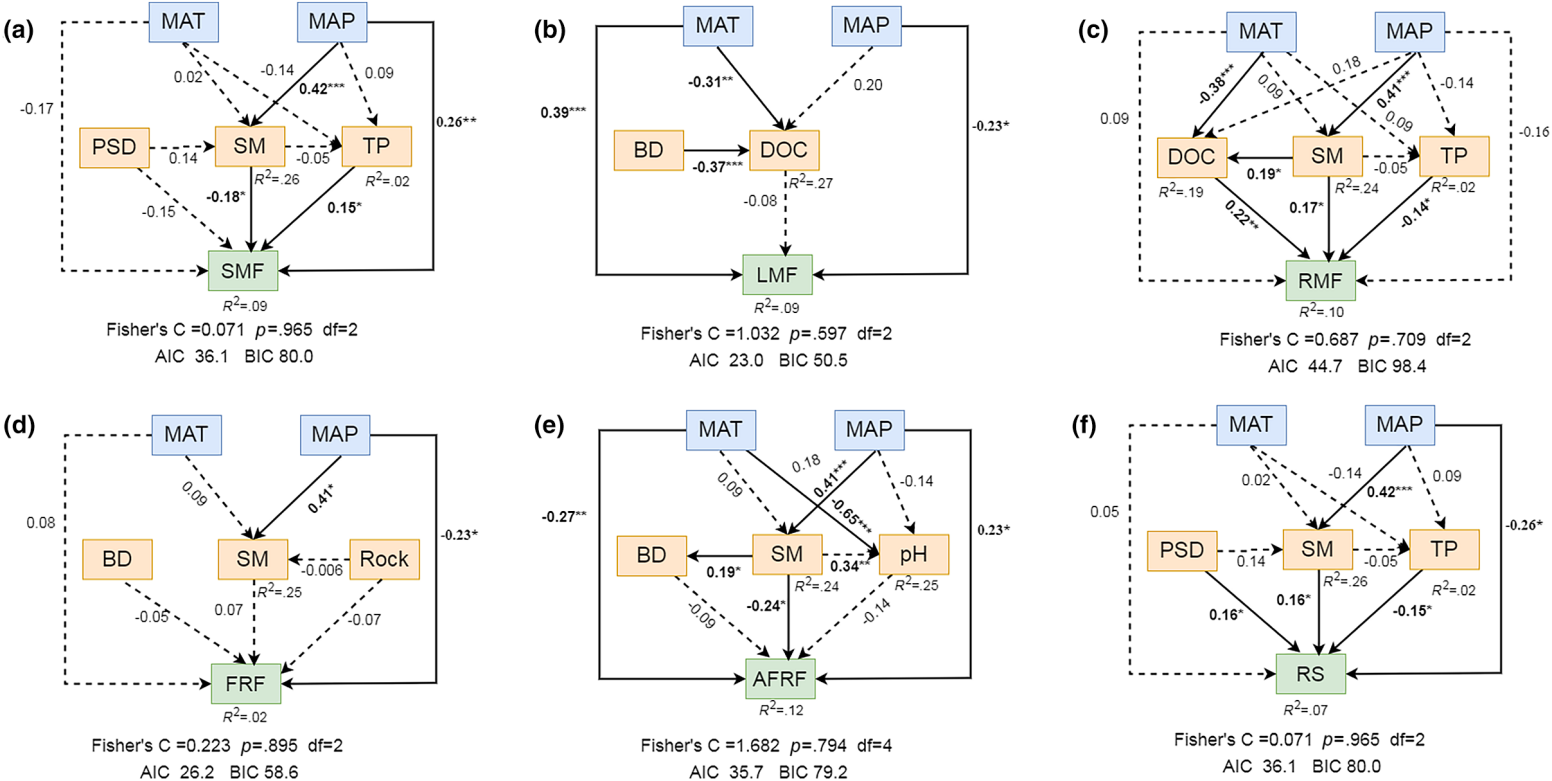
Piecewise structural equation model (piecewiseSEM) of environmental factors on biomass allocation fractions along the latitudinal gradient in dry valleys of southwest China. Full line arrows represent significant coefficient, asterisks indicate significant path (*p* < .05), * *p* < .05, **p < .01, ***p < .001, imaginary line arrows represent non‐significant coefficient (*p* > .05). *R* ^2^ indicates the extent to which other variables explain the variables as a whole. See abbreviations in Figure 5.

The results of path models showed that MAT and MAP directly affected biomass fractions except for FRF (Figure 6). In addition, MAP indirectly influenced SMF, RMF, AFRF, and R/S through positive and direct effects on soil moisture. Further, soil moisture directly and negatively influenced SMF and AFRF (Figure 6a,e), but it both RMF and R/S positively (Figure 6c,f). Soil phosphorus content also directly and positively impacted SMF, but negatively influenced RMF and R/S (Figure 6a,c,f).

**FIGURE 7:**
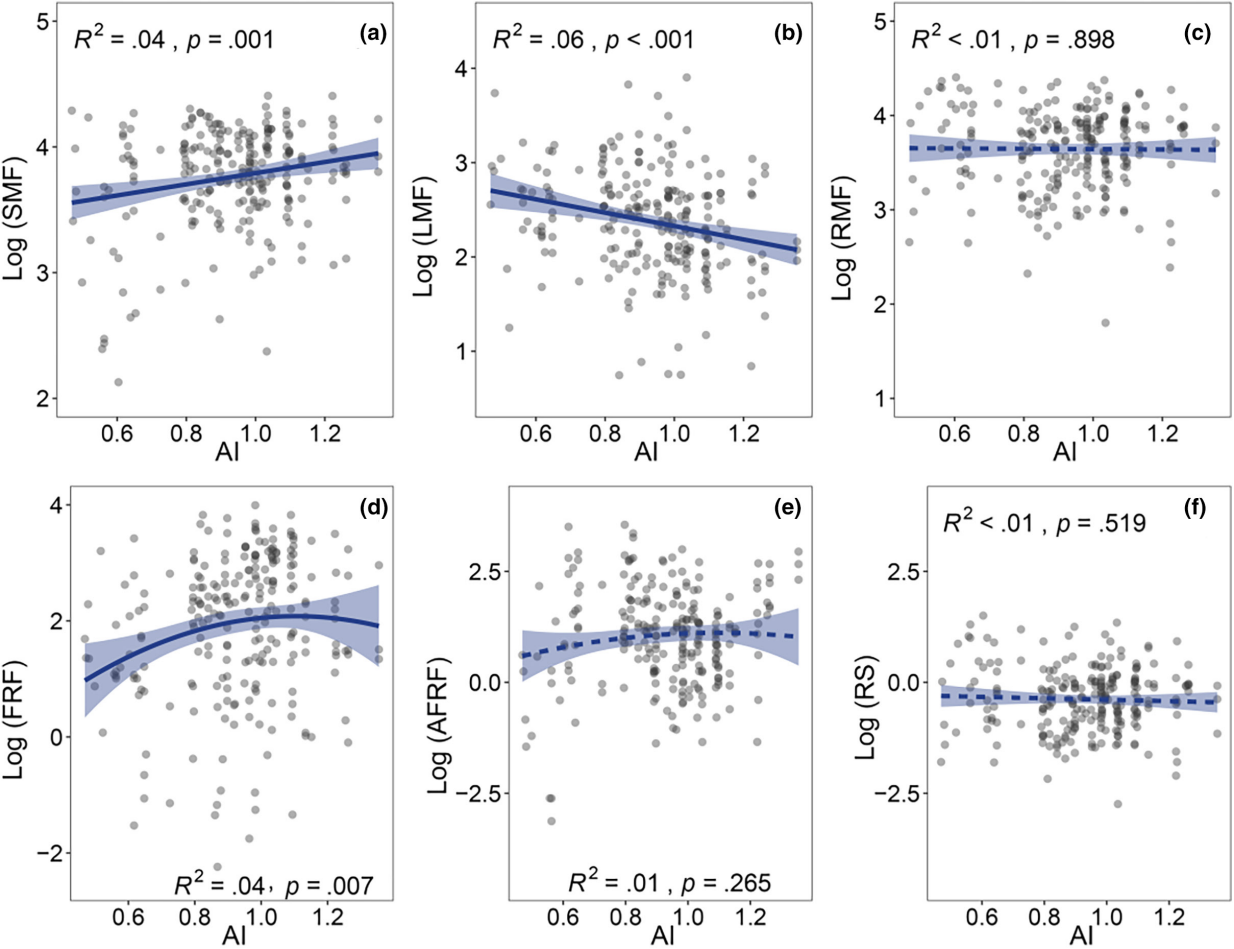
Relationships between plant biomass allocation fractions and climatic aridity index in dry valleys of southwest China. Aridity index: AI, the ratio of precipitation to evaporation. See other abbreviations in Figure 2.

In addition, with increasing aridity index, SMF showed an increasing trend, but LMF declined in the contrary (Figure 7a,b). No significant relationship was found between aridity index and RMF (Figure 7c). However, FRF exhibited a weak unimodal pattern with the change of aridity index (high aridity index represents relatively more humid climate) (Figure 7d).

## 4 DISCUSSION

Our study revealed diverse latitudinal patterns of biomass allocation fractions of different plant components in dry valleys. Moreover, we found a trade‐off relationship between biomass allocation and rooting depth which deeper root systems would have less biomass allocated to fine roots. Climatic and soil factors drive variation in different latitudinal patterns of functional biomass allocation along gradient.

### 4.1 Biomass allocation fractions across latitudinal gradients

Significant linear latitudinal patterns were found in SMF and LMF, but non‐linear latitudinal patterns were presented in FRF, AFRF, and R/S. The latitudinal patterns in our study differed with forest ecosystems, in which SMF decreased with latitude and LMF showed no latitudinal pattern across forests (Fang et al., 2018). For root systems, we did not find an obvious latitudinal pattern of RMF in dry valleys. Compared to other studies, RMF increased along the increasing latitude (De Frenne et al., 2013), but no obvious change of fine root biomass was found at the latitude from 60° N to 69° N (Finér et al., 2019). These comparisons further indicated that regional diversity of latitudinal patterns of biomass allocation varied in different ecosystems at a regional scale (Huang et al., 2021; Ma et al., 2021). However, nonlinear latitudinal patterns occurred in FRF and AFRF in our study. Because the function of root system was heterogeneous, only some of fine roots having absorptive capacity, the geographical pattern of root biomass allocation was not so obvious at a large scale (Guo et al., 2008). Therefore, plants can redistribute their biomass into different organs or functional parts as a key adaptive strategy to environmental change. In this study, for example, unimodal patterns in FRF and AFRF were consistent with soil carbon and nitrogen along the latitudinal gradient, indicating the adaptation of fine roots or absorptive roots to soil fertility, which corresponded to previous findings in dry valleys (Li et al., 2023; Yang et al., 2022, 2023).

### 4.2 Relationships of plant size and biomass allocation fractions

Plant size was recognized as a major factor in shaping biomass partitioning in plant ontogeny (Poorter et al., 2015). In our study, RMF and SMF varied mainly with PC1, showing a trade‐off relationship between RMF and SMF, meaning a high root fraction at the expense of stem growth under resource stress (Poorter et al., 2015; Umana et al., 2021). The co‐variation between SMF and plant height was favored to compete for more light resource, while the negative relationship between LMF and plant height might be due to more biomass allocated to plant shoots (Poorter et al., 2012). The larger plant size, the more biomass to absorptive roots allocated, indicating the strategy adopted by plants for increasing their absorptive capacity to meet the demands of growth and development.

Additionally, a trade‐off relationship between mass and rooting space was found in this study. A species with deeper root system would have less biomass allocated to its fine roots. The spreading extension of lateral roots and the maximum rooting depth determined the competitive capacity for soil resources with neighbors (Tumber‐Dávila et al., 2022). In arid or semi‐arid habitats plants with long and deep roots prefer to allocate limited biomass to fine roots, because plants with deeper roots are associated with a conservative strategy; these plants could absorb water from deep soil layers rather than quickly acquire water in shallow layers during raining events (Schenk & Jackson, 2002; Yang et al., 2021; Zhou et al., 2020). Thus, the trade‐off relationship allowed plants with more absorptive mass fractions to absorb more resources from the least exploited soil horizons (Freschet et al., 2018). In addition, we found that the biomass fraction of absorptive roots was independent of the architecture of root system, which mainly varied with environmental conditions rather than the ontogeny of plants.

### 4.3 Drivers of biomass allocation change along latitudinal gradient

Soil moisture was the main limiting factor in forming biomass allocation fractions except for LMF. MAT is the most important predictor of LMF, which high value of LMF is closely associated with high temperature (Reich et al., 2014). The optimal partitioning theory suggests greater biomass would be allocated to roots in both cold and dry climates (Bloom et al., 1985; Poorter et al., 2015). The increasing tendency of RMF with water availability was found in our study, this is possibly due to the significant negative influence of soil phosphorus for which nutrient limitation mainly drove the increasing investment in below‐ground biomass (Figure S2c; Yan et al., 2016; Ma et al., 2021). However, our finding further supported optimal allocation theory based on functional redistribution of root biomass resources, where plants invested more biomass in their absorptive roots to compensate for less water availability in dry valleys.

In addition, in a dry environment such as dry valleys plants allocated significantly more biomass to their leaves than in a relatively humid environment, which states that plants might invest more biomass in leaves for greater photosynthetic capacity to improve carbon investment in root system (Markesteijn & Poorter, 2009; Yin et al., 2019). The weak nonlinear pattern of FRF with aridity index indicated that the maximum allocation of fine‐root biomass occurs in relatively moderately humid areas of dry valleys. We speculated that the remission of drought stress would be favored for the investment in fine roots, through which plants could gain more resources in a relatively humid environment or raining events (Schenk & Jackson, 2002; Tomlinson et al., 2012). This pattern indicated a flexible rooting strategy for biomass allocation to increase water acquisition in a water‐limited habitat. These findings could be attributed to the complexity and vagueness of functional composition of coarse and fine roots. Therefore, more studies in various root biomass fractions could be beneficial to accurately predict the underground redistribution of root biomass resources in the future.

## 5 CONCLUSION

Our study highlighted the drivers in forming the latitudinal pattern of biomass allocation and the trade‐offs among different functional components of shrub root system in the dry valleys. It was found that the species with deeper root systems would have less biomass allocation to fine roots, showing a trade‐off relationship between biomass allocation and rooting depth. Soil moisture also significantly influenced the biomass allocation to different functional components of roots. Mean annual precipitation is the main climatic factor for forming latitudinal patterns of biomass allocation by directly influencing soil moisture and nutrients. Fine root is the key functional component for plants to adapt to drought stress and a moderate selection pressure of drought would favor the investment into fine roots.

Overall, this study filled the knowledge gap about biomass allocation in shrub root systems in dry valleys and its variation along a biogeographic gradient. Finally, we would like once again to emphasize the importance of key functional components of plants such as absorptive roots in response to environmental changes including global climate change of natural vegetation, such as absorptive roots. The biomass allocation of absorptive roots could not be ignored for species selection in ecological restoration. Furthermore, the detailed data on biomass allocation of xerophytic shrubs in dry valleys could provide more accurate information for estimating carbon cycle, particularly below‐ground carbon storage. Additionally, variations in biomass allocation patterns of shrubs were usually influenced by environments, species difference, and competition. In the future, more attention should be paid to the inter‐species and intra‐species competition for a better understanding of the effects of biological factors on biomass allocation fractions.

## AUTHOR CONTRIBUTIONS

**Yu Yang:** Conceptualization (equal); data curation (equal); formal analysis (equal); writing – original draft (equal); writing – review and editing (equal). **Zilong Wang:** Conceptualization (equal); data curation (equal); writing – review and editing (equal). **Weikai Bao:** Conceptualization (equal); funding acquisition (equal); methodology (equal); writing – review and editing (equal). **Ning Wu:** Methodology (equal); writing – review and editing (equal). **Hui Hu:** Data curation (equal); methodology (equal). **Tinghui Yang:** Data curation (equal). **Xiaojuan Li:** Data curation (equal). **Deborah Traselin Nkrumah:** Writing – review and editing (equal). **Fanglan Li:** Conceptualization (equal); data curation (equal); funding acquisition (equal); methodology (equal); writing – original draft (equal); writing – review and editing (equal).

## Supporting information


Data S1.

## Data Availability

The data that supports the findings of this study are available in the supplementary material of this article.
