# Supplementary material for: Environmental aridity driving latitudinal pattern of biomass allocation fractions in root systems of 63 shrub species in dry valleys
Source: Ecol Evol. 2024 Aug 7;14(8):e70091. doi: 10.1002/ece3.70091 (PMC11303844; doi:10.1002/ece3.70091)
Supplement: Supplementary file 1 — Data S1. [file ECE3-14-e70091-s001.docx]

**Table S1** The information of location and geographic site about sampling plot.

| Plot ID | Basin | City name | Longitude | Latitude | Elevation | Slope |
| --- | --- | --- | --- | --- | --- | --- |
| 1 | Jinsha river | Huaping | 101.404 | 26.332 | 1026 | 22.7° |
| 2 | Jinsha river | Huaping | 101.532 | 26.265 | 1024 | 40° |
| 3 | Jinsha river | Huaping | 101.260516 | 26.463 | 1136 | 34.7° |
| 4 | Jinsha river | Huaping | 101.279177 | 26.377471 | 1144 | 24.7° |
| 5 | Jinsha river | Huaping | 101.500864 | 26.40386 | 1015 | 27.3° |
| 6 | Jinsha river | Yanbian | 101.491266 | 26.394454 | 1007 | 28.3° |
| 7 | Jinsha river | Yanbian | 101.491915 | 26.392814 | 1003 | 27.6° |
| 8 | Jinsha river | Yanbian | 101.480741 | 26.371226 | 1040 | 33° |
| 9 | Jinsha river | Yanbian | 101.483569 | 26.373519 | 999 | 48.4° |
| 10 | Yuanjiang River | Yuanmou | 101.491611 | 26.511981 | 1108 | 30.4° |
| 11 | Yuanjiang River | Yuanmou | 101.492515 | 25.512590 | 1127 | 11.1° |
| 12 | Yuanjiang River | Yuanmou | 101.4925 | 25.51259 | 1127 | 24.4° |
| 13 | Yuanjiang River | Yuanmou | 101.493021 | 25.511021 | 1115 | 37.2° |
| 14 | Yuanjiang River | Yuanmou | 101.493028 | 25.511148 | 1079 | 21.3° |
| 15 | Yuanjiang River | Yuanmou | 101.514785 | 25.311189 | 1269 | 32.4° |
| 16 | Yuanjiang River | Yuanmou | 101.51454 | 25.371315 | 1300 | 33.9° |
| 17 | Yuanjiang River | Yuanmou | 101.514366 | 25.371118 | 1315 | 24.7° |
| 18 | Yuanjiang River | Yuanmou | 101.514442 | 25.370904 | 1313 | 29.5° |
| 19 | Yuanjiang River | Yuanmou | 101.480985 | 25.560455 | 1184 | 45.5° |
| 20 | Yuanjiang River | Yuanmou | 101.464581 | 25.562047 | 1231 | 18.7° |
| 21 | Yuanjiang River | Yuanmou | 101.464186 | 25.560094 | 1216 | 16.4° |
| 22 | Yuanjiang River | Yuanjiang | 102.102009 | 23.282539 | 462 | 19.2° |
| 23 | Yuanjiang River | Yuanjiang | 102.103608 | 23.282867 | 571 | 7.1° |
| 24 | Yuanjiang River | Yuanjiang | 102.105018 | 23.281766 | 486 | 9.5° |
| 25 | Yuanjiang River | Yuanjiang | 102.113254 | 23.280171 | 578 | 14.1° |
| 26 | Yuanjiang River | Yuanjiang | 102.110728 | 23.28113 | 529 | 39.1° |
| 27 | Yuanjiang River | Yuanjiang | 102.338185 | 23.38533 | 363 | 31.3° |
| 28 | Yuanjiang River | Yuanjiang | 102.335298 | 23.383236 | 349 | 32.7° |
| 29 | Yuanjiang River | Yuanjiang | 102.203101 | 23.23206 | 374 | 32.2° |
| 30 | Yuanjiang River | Yuanjiang | 102.202165 | 23.231592 | 376 | 45.8° |
| 31 | Yuanjiang River | Yuanjiang | 102.202707 | 23.23572 | 386 | 38.3° |
| 32 | Yuanjiang River | Yuanjiang | 102.194035 | 23.241732 | 378 | 23.1° |
| 33 | Yuanjiang River | Yuanjiang | 102.122785 | 23.27375 | 432 | 25.7° |
| 34 | Yuanjiang River | Yuanjiang | 102.19313 | 23.242024 | 388 | 38.5° |
| 35 | Yuanjiang River | Yuanjiang | 102.550157 | 23.401369 | 409 | 47.8° |
| 36 | Yuanjiang River | Yuanjiang | 101.551726 | 23.395619 | 410 | 42.3° |
| 37 | Yuanjiang River | Yuanjiang | 101.552108 | 23.394203 | 403 | 53.2° |
| 38 | Dadu River | Shimian | 102.418771 | 29.150306 | 1063 | 33° |
| 39 | Dadu River | Shimian | 102.172889 | 29.150617 | 899 | 40° |
| 40 | Dadu River | Shimian | 102.165993 | 29.154363 | 987 | 39° |
| 41 | Dadu River | Shimian | 102.292816 | 29.163944 | 984 | 41.5° |
| 42 | Dadu River | Shimian | 102.295303 | 29.170106 | 981 | 44.6° |
| 43 | Dadu River | Shimian | 102.302037 | 29.172238 | 991 | 37.4° |
| 44 | Dadu River | Shimian | 102.430238 | 29.248006 | 913 | 38° |
| 45 | Dadu River | Luding | 102.121903 | 29.442243 | 1257 | 34° |
| 46 | Dadu River | Luding | 102.174779 | 29.445537 | 1246 | 36.7° |
| 47 | Dadu River | Luding | 102.133972 | 29.462548 | 1316 | 47.5° |
| 48 | Dadu River | Luding | 102.135101 | 29.474368 | 1360 | 42.7° |
| 49 | Dadu River | Luding | 102.125369 | 29.485987 | 1305 | 24.3° |
| 50 | Dadu River | Luding | 102.132684 | 29.553668 | 1549 | 44.1° |
| 51 | Dadu River | Luding | 102.14027 | 29.555798 | 1556 | 29° |
| 52 | Dadu River | Luding | 102.133368 | 29.560353 | 1455 | 31° |
| 53 | Dadu River | Luding | 102.135503 | 29.563432 | 1408 | 32° |
| 54 | Dadu River | Luding | 102.121544 | 29.594465 | 1417 | 35.4° |
| 55 | Dadu River | Luding | 102.104141 | 30.012192 | 1608 | 32° |
| 56 | Dadu River | Luding | 102.103985 | 30.013433 | 1537 | 28.2° |
| 57 | Dadu River | Luding | 102.104205 | 30.013641 | 1517 | 26.3° |
| 58 | Dadu River | Luding | 102.103389 | 30.021534 | 1558 | 32.5° |
| 59 | Dadu River | Luding | 102.100877 | 30.08086 | 1548 | 35.1° |
| 60 | Dadu River | Danba | 101.555765 | 30.500521 | 1905 | 29° |
| 61 | Dadu River | Danba | 101.550796 | 30.512955 | 1869 | 20.3° |
| 62 | Dadu River | Danba | 101.514158 | 30.532948 | 1932 | 18.3° |
| 63 | Dadu River | Danba | 101.523991 | 30.551329 | 1876 | 20.2° |
| 64 | Dadu River | Danba | 101.525057 | 30.562141 | 1887 | 16.7° |
| 65 | Dadu River | Danba | 101.520077 | 31.020881 | 2121 | 41.2° |
| 66 | Minjiang River | Wenchuan | 103.301641 | 31.220719 | 1274 | 33.2° |
| 67 | Minjiang River | Wenchuan | 103.293754 | 31.22149 | 1375 | 40.2° |
| 68 | Minjiang River | Wenchuan | 103.320751 | 31.245755 | 1316 | 38.6° |
| 69 | Minjiang River | Wenchuan | 103.32196 | 31.250955 | 1385 | 35.8° |
| 70 | Minjiang River | Wenchuan | 103.381318 | 31.283119 | 1584 | 37.5° |
| 71 | Minjiang River | Wenchuan | 103.382668 | 31.293521 | 1622 | 39.9° |
| 72 | Minjiang River | Wenchuan | 103.322793 | 31.311955 | 1402 | 26.3° |
| 73 | Minjiang River | Wenchuan | 103.305955 | 31.321349 | 1489 | 28.8° |
| 74 | Minjiang River | Wenchuan | 103.304658 | 31.323132 | 1516 | 27.3° |
| 75 | Minjiang River | Wenchuan | 103.305063 | 31.331112 | 1498 | 36.1° |
| 76 | Minjiang River | Wenchuan | 103.311024 | 31.334752 | 1631 | 35.8° |
| 77 | Minjiang River | Wenchuan | 103.314687 | 31.334926 | 1582 | 38.5° |
| 78 | Minjiang River | Miaoxian | 103.522191 | 31.420022 | 1706 | 43.8° |
| 79 | Minjiang River | Miaoxian | 103.511071 | 31.422726 | 1584 | 27.8° |
| 80 | Minjiang River | Miaoxian | 103.510873 | 31.424443 | 1602 | 36° |
| 81 | Minjiang River | Miaoxian | 103.383331 | 31.493366 | 1710 | 26.3° |
| 82 | Minjiang River | Miaoxian | 103.361324 | 31.501354 | 1733 | 27.9° |
| 83 | Minjiang River | Miaoxian | 103.420968 | 31.503095 | 1801 | 35.5° |
| 84 | Minjiang River | Miaoxian | 103.457726 | 31.503543 | 1727 | 39.3° |
| 85 | Minjiang River | Miaoxian | 103.403301 | 31.504138 | 1704 | 44.6° |
| 86 | Minjiang River | Miaoxian | 103.455338 | 31.506049 | 1581 | 33.7° |
| 87 | Minjiang River | Miaoxian | 103.352222 | 31.514202 | 1738 | 34.3° |
| 88 | Minjiang River | Songpan | 103.432133 | 32.214591 | 2652 | 31.5° |
| 89 | Minjiang River | Songpan | 103.432845 | 32.220071 | 2531 | 28.9° |
| 90 | Minjiang River | Songpan | 103.433377 | 32.232341 | 2549 | 26.3° |
| 91 | Minjiang River | Songpan | 103.431196 | 32.234094 | 2611 | 32.6° |
| 92 | Minjiang River | Songpan | 103.411812 | 32.260647 | 2685 | 31.0° |

**Table S2** The base information of sampling species per family

| Species | Genus | Family | N | Site |
| --- | --- | --- | --- | --- |
| *Abelia biflora* | Abelia | Caprifoliaceae | 3 | 45;55;70 |
| *Ajania pallasiana* | Ajania | Compositae | 19 | 60;61;87;82;81;85;83;84;86;80;79;78;90;77;75;73;74;72;71 |
| *Amygdalus tangutica* | Amygdalus | Rosaceae | 3 | 88;89;92 |
| *Artemisia yunnanensis* | Artemisia | Compositae | 1 | 51;63;80 |
| *Artemisia vestita* | Artemisia | Compositae | 10 | 61;63;86;91;92;73;89;88;90;66 |
| *Barleria cristata* | Barleria | Acanthaceae | 2 | 52;53 |
| *Bauhinia brachycarpa* | Bauhinia | Leguminosae | 17 | 60;61;63;47;49;50;48;53;56;58;59;83;2;43;73;5;49 |
| *Berberis wilsoniae* | Berberis | Berberidaceae | 1 | 91 |
| *Buxus sinica* | Buxus | Buxaceae | 2 | 37;73 |
| *Campylotropis macrocarpa* | Campylotropis | Leguminosae | 4 | 51;68;73;77 |
| *Campylotropis wilsonii* | Campylotropis | Leguminosae | 13 | 87;82;81;85;86;79;78;76;75;74;72;66;70 |
| *Caragana franchetiana* | Caragana | Leguminosae | 6 | 54;64;86;78;66;68 |
| *Caryopteris forrestii* | Caryopteris | Lamiaceae | 1 | 77 |
| *Caryopteris glutinosa* | Caryopteris | Lamiaceae | 6 | 87;82;81;85;83;79 |
| *Ceratostigma minus* | Ceratostigma | Plumbaginaceae | 8 | 56;57;60;61;64;71;76;84 |
| *Ceratoides latens* | Ceratoides | Chenopodiaceae | 4 | 80;88;92;69 |
| *Clausena excavata* | Clausena | Rutaceae | 2 | 24;29 |
| *Cotinus szechuanensis* | Cotinus | Anacardiaceae | 4 | 67;69;70;78 |
| *Cotoneaster submultiflorus* | Cotoneaster | Rosaceae | 2 | 91;92 |
| *Croton yunnanensis* | Croton | Euphorbiaceae | 3 | 56;58;59 |
| *Cynanchum wilfordii* | Cynanchum | Apocynaceae | 1 | 60 |
| *Dalbergia obtusifolia* | Dalbergia | Leguminosae | 1 | 17 |
| *Daphne gemmata* | Daphne | Thymelaeaceae | 8 | 16;78;90 |
| *Desmodium multiflorum* | Desmodium | Leguminosae | 10 | 50;53;54;46;86;39;44;72;66;67 |
| *Diospyros dumetorum* | Diospyros | Ebenaceae | 2 | 46;49 |
| *Dodonaea viscosa* | Dodonaea | Sapindaceae | 8 | 4;10;12;20;28;29;45;54 |
| *Elsholtzia ciliata* | Elsholtzia | Labiatae | 2 | 51;64 |
| *Flemingia macrophylla* | Flemingia | Leguminosae | 3 | 6;11;21 |
| *Grewia biloba* | Grewia | Tiliaceae | 2 | 15; 64 |
| *Indigofera amblyantha* | Indigofera | Leguminosae | 4 | 52;53;79;80 |
| *Indigofera bungeana* | Indigofera | Leguminosae | 3 | 42;82;84 |
| *Jasminum nudiflorum* | Jasminum | Oleaceae | 3 | 66;68;71 |
| *Jasminum yuanjiangense* | Jasminum | Oleaceae | 1 | 27 |
| *Krascheninnikovia ceratoides* | Krascheninnikovia | Amaranthaceae | 1 | 85 |
| *Leptodermis buxifolia* | *Leptodermis* | Rubiaceae | 2 | 82;87 |
| *Lespedeza cuneata* | Lespedeza | Fabaceae | 2 | 74;77 |
| *Leptodermis pilosa* | Leptodermis | Rubiaceae | 3 | 38;50;51 |
| *Lespedeza bicolor* | Lespedeza | Leguminosae | 4 | 7;38;43;48 |
| *Leptodermis schneideri* | Leptodermis | Rubiaceae | 3 | 38;56;58 |
| *Lindera glauca* | Lindera | Lauraceae | 1 | 44 |
| *Myrsine africana* | Myrsine | Myrsinaceae | 3 | 51;65;69 |
| *Nouelia insignis* | Nouelia | Asteraceae | 1 | 16 |
| *Osteomeles anthyllidifolia* | Osteomeles | Rosaceae | 1 | 65 |
| *Ostryopsis davidiana* | Ostryopsis | Betulaceae | 1 | 70 |
| *Osyris wightiana* | Osyris | Santalaceae | 2 | 19;64 |
| *Polyalthia cerasoides* | Polyalthia | Annonaceae | 1 | 22 |
| Pulicaria chrysanth | Pulicaria | Asteraceae | 1 | 74 |
| *Phyllanthus emblica* | Phyllanthus | uphorbiaceae | 1 | 8 |
| *Pistacia weinmannifolia* | Pistacia | Anacardiaceae | 2 | 41;43 |
| *Rabdosia amethystoides* | Rabdosia | Labiatae | 12 | 60;63;87;82;84;86;79;78;90;91;71;18 |
| *Rhamnus davurica* | Rhamnus | Rhamnaceae | 2 | 44;69 |
| *Rubus niveus* | *Rubus* | Rosaceae | 1 | 51 |
| Rhus chinensis | Rhus | Anacardiaceae | 1 | 41 |
| *Sageretia pycnophylla* | Sageretia | Rhamnaceae | 1 | 50 |
| *Sophora davidii* | Sophora | Leguminosae | 5 | 61;62;63;84;70 |
| *Spiraea salicifolia* | Spiraea | Rosaceae | 2 | 71;90 |
| *Spiraea wilsonii* | Spiraea | Rosaceae | 1 | 92 |
| *Tarenna depauperata* | Tarenna | Rubiaceae | 1 | 26 |
| *Vitex negundo* | Vitex | Verbenaceae | 17 | 1;3;9;13; 27;42;57;58;59;24;25;30;31;32;33;34;36 |
| *Viburnum congestum* | Viburnum | Viburnaceae | 3 | 40;43;69 |
| *Vitis flexuosa* |  |  |  |  |
| *Wikstroemia canescens* | Wikstroemia | Thymelaeaceae |  | 61;76;77;75;74; |
| *Zanthoxylum stenophyllum* | Zanthoxylum | Rutaceae | 2 | 62;64 |

**Table S3** The phylogenetic signal ( Blomberg’s K and Pagel’s Lambda ) among different biomass allocation fractions. **SMF**: stem mass fraction; **LMF**: leaf mass fraction; **RMF**: root mass fraction; **FRF**: fine-root mass fraction; **AFRF**: absorptive root mass fraction.

| **Biomass** | **K** | **P** | **λ** | **P** |
| --- | --- | --- | --- | --- |
| SMF | 0.19 | 0.40 | 0.13 | 0.37 |
| LMF | 0.37 | 0.11 | <0.001 | 1 |
| RMF | 0.29 | 0.07 | <0.001 | 1 |
| FRF | 0.18 | 0.48 | <0.001 | 1 |
| AFRF | 0.38 | 0.07 | 0.87 | 0.15 |


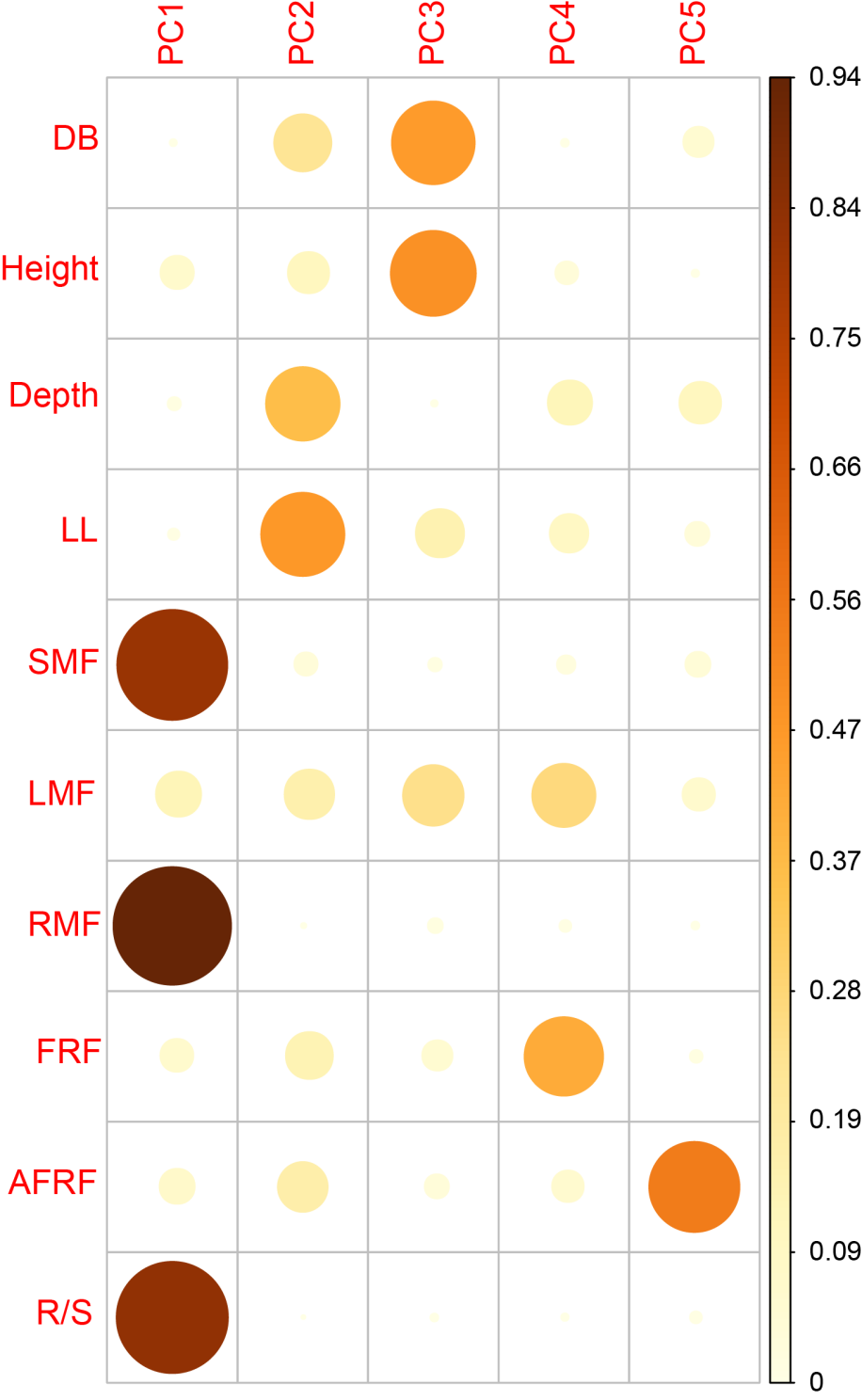


**Figure S1** The loading values of biomass allocation fractions of principal components analysis (PCA). **DB**: basal diameter; **Height**: plant height; **Depth**: root depth; **LL**: Lateral root extension length; **SMF**: stem mass fraction; **LMF**: leaf mass fraction; **RMF**: root mass fraction; **FRF**: fine-root mass fraction; **AFRF**: absorptive root mass fraction; **R/S**: root-shoot ratio.


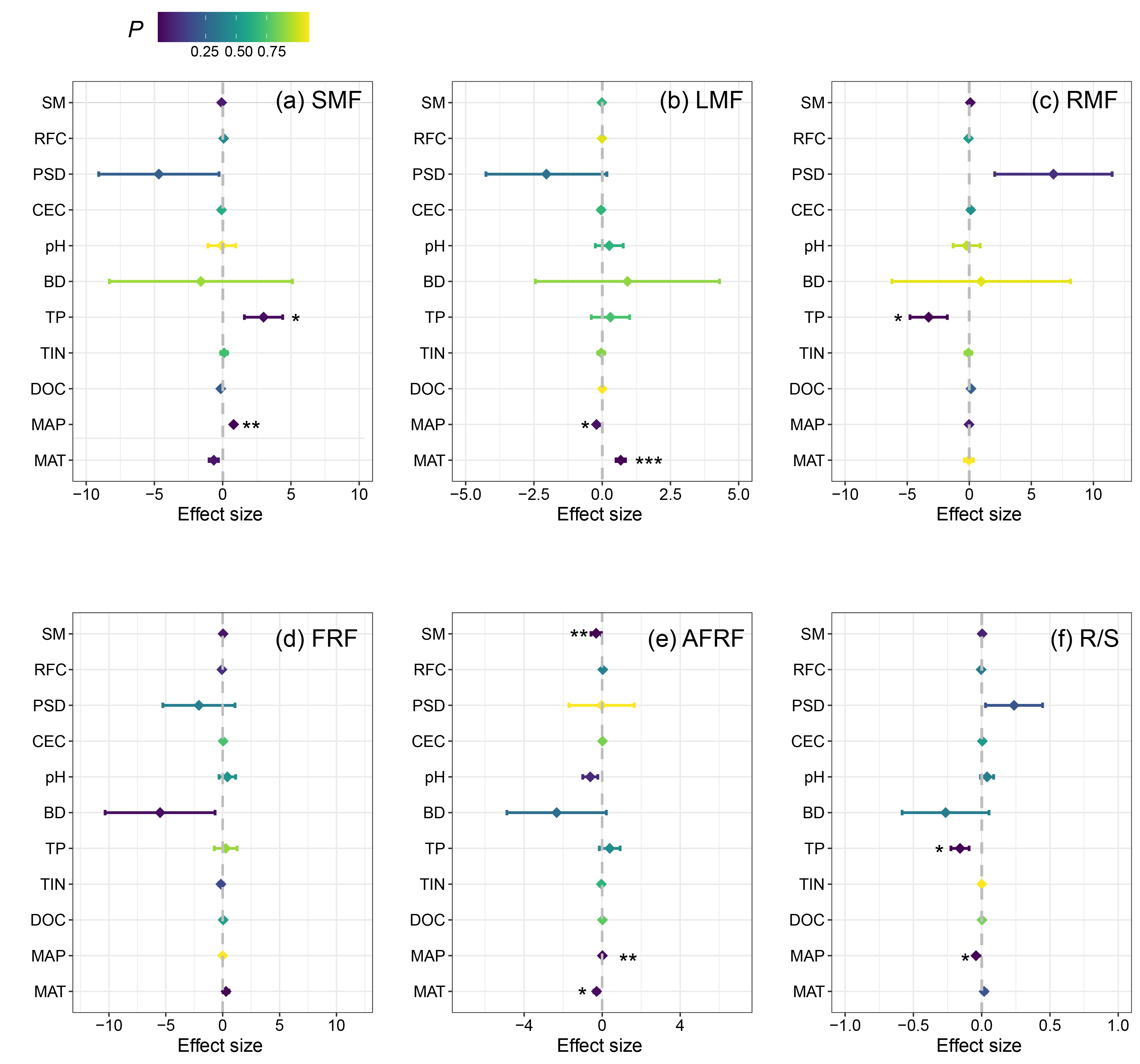


**Figure S2** Effect of climatic and soil factors on biomass allocation fraction base on linear mixed-effects models. **SMF**: stem mass fraction; **LMF**: leaf mass fraction; **RMF**: root mass fraction; **FRF**: fine-root mass fraction; **AFRF**: absorptive root mass fraction; **R/S**: root-shoot ratio; **BD**: bulk density; **DOC**: dissolved organic carbon; **MAT**: Mean annual temperature; **MAP**: mean annual precipitation; **PSD**: particle size distribution; **pH**: pondus hydrogenii; **RFC**: rock fragment content; **SM**: soil moisture; **TIN**: total inorganic nitrogen. Asterisks indicate significant path, * *P*<0.05, ***P*<0.01, ****P*<0.001.
